# Acknowledgement to the reviewers of GMS Journal for Medical Education

**DOI:** 10.3205/zma001656

**Published:** 2024-02-15

**Authors:** Martin R. Fischer, Götz Fabry

**Affiliations:** 1 GMS Journal for Medical Education, Chief Editor, Erlangen, Germany; 2 University Hospital, LMU Munich, Institute for Medical Education, Munich, Germany; 3 Albert-Ludwig-Universität Freiburg, Institut für Medizinische Psychologie und Soziologie, Freiburg, Germany

## Acknowledgement

We gratefully acknowledge the valuable contribution of the following peer reviewers, who supported our Journal in 2023. We are looking forward to continuing our collaboration!

## Reviewers in alphabetical order

- Kambiz Afshar, Hannover
- Olaf Ahlers, Berlin
- Matthias Angstwurm, München
- Jana Aulenkamp, Essen
- Katrin Balzer, Lübeck
- Ivan Bank, Amsterdam (Niederlande)
- Daniel Bauer, Bern (Schweiz)
- Nicola Bauer, Köln
- Adrian Baumann, München
- Michael Bedek, Graz (Österreich)
- Silke Biller, Basel (Schweiz)
- Kerstin Bitter, Halle/Saale
- Sebastian Bode, Ulm
- Christoph Bohne, Frankfurt/Main
- Barbara Braun, Mannnheim
- Jan Breckwoldt, Zürich (Schweiz)
- Irene Brunk, Berlin
- Johanna Büchel, München
- Stefan Bushuven, Radolfzell
- Jean-Francois Chenot, Greifswald
- Iris Demmer, Göttingen
- Andreas Dinkel, München
- Sabine Drossard, Würzburg
- Fabian Dupont, Homburg
- Jan P. Ehlers, Witten
- Maren Ehrhardt, Hamburg
- Rebecca Sara Erschens, Tübingen
- Teresa Festl-Wietek, Tübingen
- Sabine Ingrid Fischbeck, Mainz
- Volkhard Fischer, Hannover
- Kristina Flägel, Lübeck
- Michael Freitag, Oldenburg
- Julia Freytag, Berlin
- Olaf Fritze, Tübingen
- Robert Gaschler, Hagen
- Shewatatek Gedamu, Jimma (Äthiopien)
- Kirsten Gehlhar, Oldenburg
- Sabine Gehrke-Beck, Berlin
- Waltraud Georg, Augsburg
- Marianne Giesler, Freiburg im Breisgau
- Katharina Glassen, Heidelberg
- Erik Oliver Glocker, Freiburg im Breisgau
- Heide Götze, Leipzig
- Gertraud Gradl-Dietsch, Essen
- Joachim Graf, Tübingen
- Tanja Graupe, München
- Dennis Grevenstein, Heidelberg
- David Groneberg, Frankfurt/Main
- Markus Gulich, Ulm
- David Gurrea Salas, Windisch
- Maximiliane Hädicke, Göttingen
- Eckhart G. Hahn, Erlangen
- Laura Hanke, Mainz
- Roman Hari, Bern (Schweiz)
- Patrick Heger, Ulm
- Andrea Heinzmann, Freiburg im Breisgau
- Eva Hennel, Bern (Schweiz)
- Marit Herbolzheimer, Murnau
- Stephanie Herbstreit, Essen
- Monika Himmelbauer, Wien (Österreich)
- Barbara Hinding, Mainz
- Anke Hollinderbäumer, Mainz
- Ylva Holzhausen, Berlin
- Christopher Holzmann-Littig, München
- Angelika Homberg, Mannheim
- Luke Hopf, Hamburg
- Johanna Huber, München
- Nana Jedlicska, München
- Barbara Jömann, Bochum
- Robert Jungwirth, Ulm
- Martina Kadmon, Augsburg
- Hanna Kaduszkiewicz, Kiel
- Julius Josef Kaminski, Berlin
- Verena Kantenwein, München
- Rolf Kienle, Berlin
- Claudia Kiessling, Witten
- Alexander Kiss, Basel (Schweiz)
- Christin Kleinsorgen, Hannover
- Fee Klupp, Heidelberg
- Katharina Knie, Freiburg im Breisgau
- Thomas Kollewe, Frankfurt/Mainz
- Andrzej Kononowicz, Krakow (Polen)
- Mirjam Körner, Freiburg im Breisgau
- Felix Krause, Aachen
- Susanne Kühl, Ulm
- Sandy Kujumdshiev, Leipzig
- Raphael Kunisch, Augsburg
- Nathalie Laissue, Basel (Schweiz)
- Ute Lange, Bochum
- Wolf Langewitz, Basel (Schweiz)
- Ronny Lehmann, Heidelberg
- Sven Lindner, Düsseldorf
- Sabine Ludwig, Berlin
- Ruth Mächler, München
- Frederik Mader, Nittendorf
- Jan Matthes, Köln
- David Messerer, Ulm
- Parisa Moll-Khosrawi, Hamburg
- Andreas Möltner, Heidelberg
- Matteo Monti, Lausanne (Schweiz)
- Sören Moritz, Köln
- Christiane Müller, Göttingen
- Laura Müller, Ulm
- Felix Nickel, Heidelberg
- Heidi Oberhauser, Innsbruck (Österreich)
- Cihan Papan, Bonn
- Irina Papazova, Augsburg
- Dorothea Penders, Berlin
- Andrea Petermann-Meyer, Aachen
- Harm Peters, Berlin
- Sarah Prediger, Hamburg
- Wolfgang Prodinger, Innsbruck (Österreich)
- Valentina Püschel, Mannheim
- Christina Quandt, Hannover
- Alexander Rahman, Hannover
- Florian Recker, Bonn
- Maike Reingen, Dresden
- Alexander Renkl, Freiburg im Breisgau
- Anne Röhle, Dresden
- Carolin Rolle, Augsburg
- Bernd Romeike, Rostock
- Miriam Rothdiener, Tübingen
- Emma C. Ryan, New Haven (USA)
- Thorsten Schäfer, Bochum
- Stefan Schauber, Oslo (Norwegen)
- Christian Scheffer, Witten
- Kristina Schick, München
- Simona-Georg Schick, Heidelberg
- Claudia Schlegel, Bern (Schweiz)
- Anita Schmidt, Erlangen
- Karen Schmidt-Baese, München
- Eva Schönefeld, Münster
- Renate Schramek, Bochum
- Teresa Schreckenbach, Frankfurt/Main
- Simon Schwill, Heidelberg
- Alexander Peter Schwoerer, Hamburg
- Monika Sennekamp, Frankfurt/Main
- Thomas Shiozawa-Bayer, Tübingen
- Jan Siebenbrock, Münster
- Robert Speidel, Ulm
- Babette Stadler-Werner, Regensburg
- Robin Stark, Saarbrücken
- Beate Stock-Schröer, Witten
- Jessica Stokes-Parish, Gold Coast (Australien)
- Christoph Stosch, Köln
- Ulrich Stössel, Freiburg im Breisgau
- Sylvia Stracke, Greifswald
- Ulrike Subotic, Basel (Schweiz)
- Cynthia Szalai, Mülheim a.d. Ruhr
- Julia Tabatabai, Heidelberg
- Christian Thrien, Köln
- Judith Ullmann-Moskovits, Frankfurt/Main
- Mirdita Useini, Zürich (Schweiz)
- Maria Paula Valk-Draad, Witten
- Ivo Volf, Wien (Österreich)
- Thomas von Lengerke, Hannover
- Felicitas L. Wagner, Bern (Schweiz)
- Yvonne Wagner, Stuttgart
- Michaela Wagner-Menghin, Wien (Österreich)
- Stefan Watzke, Halle/Saale
- Birgitta Weltermann, Bonn
- Hans-Jürgen Wenz, Kiel
- Bärbel Wesselborg, Düsseldorf
- Jürgen Westermann, Lübeck
- Simone Weyers, Düsseldorf
- Swantje Wienand, Bremen
- Solomon Worku, Addis Ababa (Äthiopien)
- Anja Zimmermann, Berlin
- Michaela Zupanic, Witten

## Authors’ ORCIDs

- Martin R. Fischer: 0000-0002-5299-5025
- Götz Fabry: 0000-0002-5393-606X

